# Supplementary material for: A macrozoobenthic data set of the Black Sea northwestern shelf
Source: Sci Data. 2025 Jun 7;12:957. doi: 10.1038/s41597-025-05311-2 (PMC12145434; doi:10.1038/s41597-025-05311-2)
Supplement: Supplementary file 1 — Supplementary Information [file 41597_2025_5311_MOESM1_ESM.pdf]

Supplementary information

Table of content

1. Handling the data 1
2. Multivariate ordination of taxa and traits 3
3. Synthesizing a combined trait indicator... 6
References.. 9

The document provides some guidelines to handle the provided trait data in R and synthesize new traits based on the provided ones.

1. Handling the data

Load the provided commands and the package ade4[1,2] for multivariate ordination.

```
Source(".../bind_traits.R")
source(".../syn_trait.R")
require(ade4)
```

Load the trait data & labels.

```
data <- read.csv("traits.csv", h = T)
lab <- read.csv("labels.csv", h = T)
head(data)
```

The trait data frame is vertically partitioned, each trait being a block of trait modality variables. The label data frame `lab` lists the successive traits coded in the column `Trait.code` and named in `Trait`. Within each trait, the column `Modality` provides the modality labels. The column `Column.name` contains the column labels of the trait data frame, hereafter called `tab` (see below).

```
head(lab, 20)

   Column.name Trait.code Modality.code                  Trait          Modality
1        T1.M1          1             1              Life span                <1
2        T1.M2          1             2              Life span               1-3
3        T1.M3          1             3              Life span              3-10
4        T1.M4          1             4              Life span             10-20
5        T1.M5          1             5              Life span               >20
6        T2.M1          2             1        Age at maturity                <1
7        T2.M2          2             2        Age at maturity               1-3
8        T2.M3          2             3        Age at maturity                >3
9        T3.M1          3             1             Sexuality       Gonochorism
10       T3.M2          3             2             Sexuality          Homogamy
11       T3.M3          3             3             Sexuality         Protandry
12       T4.M1          4             1 Reproductive frequency   Sexual seasonal
13       T4.M2          4             2 Reproductive frequency Sexual continuous
14       T4.M3          4             3 Reproductive frequency           Asexual
15       T5.M1          5             1          Fertilisation      Broadcasting
16       T5.M2          5             2          Fertilisation      Spermcasting
17       T5.M3          5             3          Fertilisation           Pairing
```

```
18       T6.M1          6             1       Annual fecundity             <10e2
19       T6.M2          6             2       Annual fecundity         10e2-10e3
20       T6.M3          6             3       Annual fecundity         10e3-10e4
...
```

Extract the trait data. `tab` is the taxa × trait modalities data frame linked to `lab` by its column names sorted in the same order.

```
data.frame(colnames(data))
tab <- data[7:124]
rownames(tab) <- data$Taxon
```

Selection of two sets of response traits. Firstly, identify them.

```
unique(lab[c("Trait.code", "Trait")])

    Trait.code                            Trait
1            1                        Life span
6            2                  Age at maturity
9            3                        Sexuality
12           4           Reproductive frequency
15           5                    Fertilisation
18           6                 Annual fecundity
24           7                    Offspring type
27           8                    Offspring size
31           9             Offspring protection
35          10            Offspring development
40          11 Offspring benthic stage duration
45          12 Offspring pelagic stage duration
50          13        Offspring settlement size
54          14                        Body mass
60          15                      Body length
65          16                         Mobility
70          17         Substratum depth occupancy
75          18          Epi-bioconstruction type
81          19     Epi-bioconstruction extension
85          20          Epi-bioconstruction size
92          21         Endo-bioconstruction type
95          22        Endo-bioconstruction depth
100         23        Endo-bioconstruction width
104         24              Ventilation/Pumping
107         25               Sediment mixing type
112         26                  Biostabilisation
115         27                      Feeding type

tab1 <- tab[lab$Trait.code %in% 15:16]
tab2 <- tab[lab$Trait.code %in% 1:13]
lab1 <- lab[lab$Trait.code %in% 15:16,]
lab2 <- lab[lab$Trait.code %in% 1:13,]
```

Bind `tab1` and `tab2` in a single data frame with an adapted label data frame by means of the command `bind.traits`.

```
w <- bind.traits(list(tab1, tab2), list(lab1, lab2))
tab.resp <- w$tab
lab.resp <- w$lab
head(tab.resp[1:10])
```

```
                           T1.M1 T1.M2 T1.M3 T1.M4 T1.M5 T2.M1 T2.M2 T2.M3 T2.M4
Abra alba                      0     1     0     0     0     0     1     0     0
Abra nitida                    0     1     0     0     0     0     1     0     0
Abra segmentum                 0     1     0     0     0     0     1     0     0
Acanthocardia paucicostata     0     0     1     0     0     0     1     1     0
Actinia equina                 0     0     1     0     0     1     0     0     0
Alitta succinea                0     0     0     1     0     0     0     1     1
                           T2.M5
Abra alba                      0
Abra nitida                    0
Abra segmentum                 0
Acanthocardia paucicostata     0
Actinia equina                 0
Alitta succinea                0
...

head(lab.resp, 50)

   Column.name Trait.code Modality.code                 Trait          Modality
1        T1.M1          1             1           Body length                <1
2        T1.M2          1             2           Body length               1-3
3        T1.M3          1             3           Body length              3-10
4        T1.M4          1             4           Body length             10-20
5        T1.M5          1             5           Body length               >20
6        T2.M1          2             1              Mobility          Immobile
7        T2.M2          2             2              Mobility           Limited
8        T2.M3          2             3              Mobility              Slow
9        T2.M4          2             4              Mobility              Fast
10       T2.M5          2             5              Mobility         Very fast
11       T3.M1          3             1             Life span                <1
12       T3.M2          3             2             Life span               1-3
13       T3.M3          3             3             Life span              3-10
14       T3.M4          3             4             Life span             10-20
15       T3.M5          3             5             Life span               >20
16       T4.M1          4             1       Age at maturity                <1
17       T4.M2          4             2       Age at maturity               1-3
18       T4.M3          4             3       Age at maturity                >3
...
```

2. Multivariate ordination of taxa and traits

Prior to ordination, the data are fuzzy coded according to[3].

```
w <- prep.fuzzy.var(tab.resp, table(lab.resp$Trait.code))
```

Fuzzy Correspondence Analysis[2].

```
fca <- dudi.fca(w, scan = F)
```

Check the dimensionality of the data with the eigenvalues.

```
barplot(fca$eig)
```

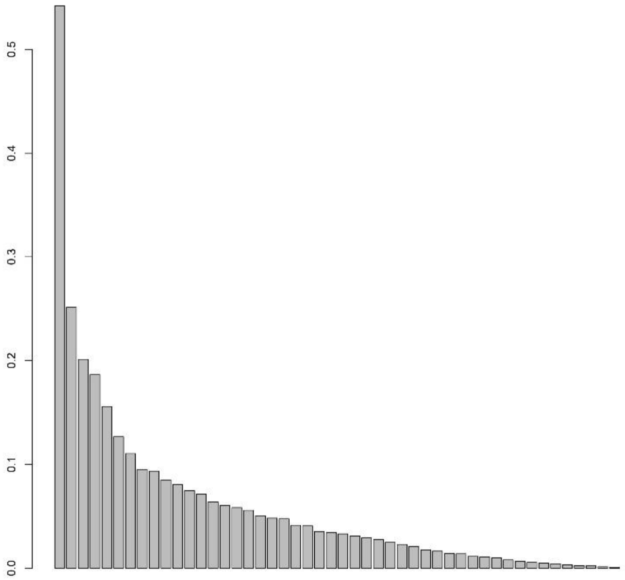

Represent the taxa (black dots) and traits (one per graphical window) along the two first axes.

```
par(mfrow = c(5, 3))
for(i in 1:15){
  s.label(fca$li, clab = 0)
  s.distri(fca$li, tab.resp[lab.resp$Trait.code == i],
           lab = lab.resp$Modality[lab.resp$Trait.code == i],
           cpoint = 2, clab = 1.5, cstar = 0,
           sub = unique(lab.resp$Trait)[i],
           possub = "topleft", csub = 2, add.p = T)
}
```

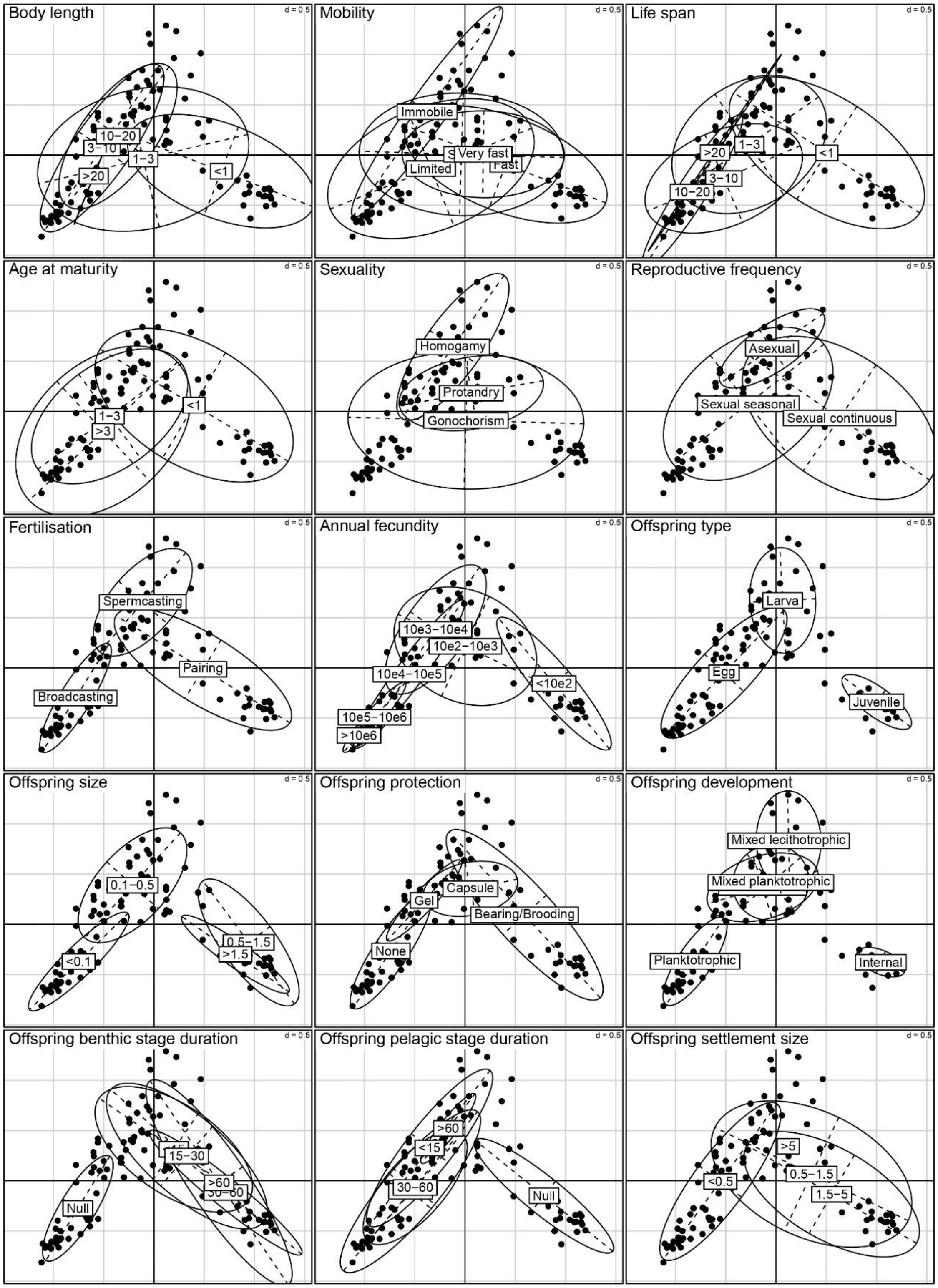

The pattern clearly highlights the three life strategies identified[4]: on the right side, stress-resistant taxa (A-strategists); on the top, disturbance-resilient taxa (r-strategists); on the left, long-lived and slow growing species typical of low hydrodynamics (K-strategists).

3. Synthesizing a combined trait indicator

The data set is composed of traits commonly described in natural history studies. However, some processes, especially ecosystem functions, require the combination of several traits so that they be relevantly expressed. The command `syn.trait` enables the combination of several traits with the desired formula. It was designed in a flexible way to specifically handle traits as blocks of trait modality variables for which transformations such as fuzzy coding and scaling can be needed to adjust trait weights in the calculation.

Bioturbation, that includes sediment biomixing types and bioirrigation[5], is a relevant example. The calculation proposed here is illustrated in Beauchard et al. (2023)[6] for several effect traits.

Biomixing. There are four different types of sediment biomixing[5,7]: biodiffusion, upward conveying, downward conveying and regeneration. They are the four last modalities of the trait 25 (the first one being "None", i.e., no affinity for none of them). Following a formula that seems to make consensus nowadays[8], here is the calculation of biodiffusive mixing potential at the species level. Trait 25:

```
lab[lab$Trait == "Sediment mixing type",]

    Column.name Trait.code Modality.code                Trait          Modality
107      T25.M1         25             1 Sediment mixing type              None
108      T25.M2         25             2 Sediment mixing type         Diffusion
109      T25.M3         25             3 Sediment mixing type   Upward conveying
110      T25.M4         25             4 Sediment mixing type Downward conveying
111      T25.M5         25             5 Sediment mixing type       Regeneration
```

We are interested in the modality "Diffusion", but also in the modality "None" as some epibenthic taxa are intermittently buried and weakly diffusive (e.g. mysids). Therefore, they are scored 1/1/0/0/0; through fuzzy coding, their diffusive ability is lower (0.5/0.5/0.0/0.0/0.0) than those also biodiffusors but permanently and more continuously buried (0.0/1.0/0.0/0.0/0.0). Hence, a new trait is specifically created:

```
w1 <- tab[lab$Trait.code == 25][1:2]
w2 <- lab[lab$Trait.code == 25,][1:2,]
w2$Trait <- rep("Biodiffusion", nrow(w2))
w <- bind.traits(list(tab, w1), list(lab, w2))
lab <- w$lab
tab <- w$tab
tail(lab, 4)


...
    Column.name Trait.code Modality.code        Trait  Modality
117      T27.M3         27             3 Feeding type      HeGr
118      T27.M4         27             4 Feeding type      CaSc
119      T28.M1         28             1 Biodiffusion      None
120      T28.M2         28             2 Biodiffusion Diffusion
```

Then, identify additional traits contributing to biodiffusive mixing rate (amount of sediment displaced in all directions per time unit).

```
unique(lab[c("Trait.code", "Trait")])
    Trait.code                        Trait
1            1                    Life span
6            2              Age at maturity
9            3                    Sexuality
```

```
12          4          Reproductive frequency
15          5                   Fertilisation
18          6                Annual fecundity
24          7                  Offspring type
27          8                  Offspring size
31          9            Offspring protection
35         10           Offspring development
40         11 Offspring benthic stage duration
45         12 Offspring pelagic stage duration
50         13       Offspring settlement size
54         14                       Body mass
60         15                     Body length
65         16                        Mobility
70         17      Substratum depth occupancy
75         18        Epi-bioconstruction type
81         19   Epi-bioconstruction extension
85         20        Epi-bioconstruction size
92         21       Endo-bioconstruction type
95         22      Endo-bioconstruction depth
100        23      Endo-bioconstruction width
104        24              Ventilation/Pumping
107        25             Sediment mixing type
112        26                 Biostabilisation
115        27                     Feeding type
119        28                     Biodiffusion
```

The formula firstly considers the affinity for biodiffusion which is trait 28 (`which.traits = c(28,...)`); the modalities of this trait are multiplied by the scores 0/1 so that only effective biodiffusive mixing be non-null (`list.sco = list(c(0,1),...`). Following this scoring procedure, the next considered traits are body mass (14), mobility (16) and substratum depth occupancy (17). These traits are multiplied by the performance score profiles 1/2/3/4/5/6 (from <0.001 to > 10.000 g AFDM), 0/1/2/3/4 (from immobile to very fast) and 1/1/1/1/1 (from 0 to > 30 cm deep), respectively. Note that the score 0 for the modality "Immobile" in mobility induces a null performance. Prior to multiplication by performance scores, the command `fuz` indicates whether the raw scores (0 or 1) are processed by fuzzy coding so that the values for a species within a trait sum to 1. Then, the sum of the values following multiplication by performance scores returns a mean trait value (i.e., non-uniformly weighted mean). In the case of the last trait (vertical distribution), `fuz` is attributed `F` since we want to express the thickness of the sediment matrix in which the organism prospects. `trait.scale = T` enables the rescaling of each trait between 0 and 1 before the calculation (the traits get the same importance), specified by `formula`. Optionally, the final synthetic trait can be rescaled between 0 and 1 with `index.scale = T`.

```
dif <- syn.trait(tab = tab, lab = lab,
                 which.traits = c(28, 14, 16, 17),
                 list.sco = list(c(0, 1),
                                 c(1:6),
                                 c(0:4),
                                 c(rep(1, 5))),
                 fuz = c(T, T, T, F),
                 fun = c("sum", "sum", "sum", "sum"),
                 formula = "x1 * x2 * x3 * x4",
                 sco.scale = T,
                 trait.scale = T,
                 index.scale = T)

sort(dif, decreasing = T)[1:30]

         Upogebia pusilla           Alitta succinea        Glycera tridactyla
```

```
              1.0000000                 0.8333333                 0.8333333
    Hediste diversicolor           Crangon crangon             Rapana venosa
              0.8333333                 0.5833333                0.5000000
Branchiostoma lanceolatum        Diogenes pugilator              Scolelepis
              0.4687500                 0.4375000                 0.4166667
       Eurydice dollfusi            Tritia neritea         Tritia reticulata
              0.3750000                 0.3333333                0.3333333
       Nephtys hombergii      Perinereis cultrifera      Phyllodoce maculata
              0.3125000                 0.3125000                 0.3125000
       Phyllodoce mucosa        Corophium volutator        Donax semistriatus
              0.3125000                 0.2500000                 0.2500000
    Anadara kagoshimensis    Leptosynapta inhaerens    Synchelidium maculatum
              0.2083333                 0.1875000                 0.1875000
          Iphinoe elisae          Iphinoe maeotica           Iphinoe tenella
              0.1666667                 0.1666667                0.1666667
       Retusa robagliana         Retusa truncatula         Retusa umbilicata
              0.1666667                 0.1666667                 0.1666667
       Retusa variabilis      Cerastoderma glaucum      Eunereis longissima
              0.1666667                 0.1562500                 0.1562500
...
```

The resulting synthetic trait (`dif`), when multiplied by organism densities (individual or biomass density) and summed across species, gives the sediment biodiffusive potential of the species community. The same can be done for the three other biomixing types (with an adapted scoring to the biomixing mechanism); the sum of the four can, if carefully scaled between 0 and 1 (`index.norm = T`), gives the total sediment biomixing potential. The bioturbation potential from[8] ("Bpc"), confusedly denominated regarding the clarification of[5], was proposed for such a measurement. Our method provides an improvement for three reasons. Firstly, a species biomixing potential is not necessarily specific to a sediment mixing type, a species can have positive affinities for several types (e.g., biodiffusive mixing when burrowing and upward conveying when defecating). Secondly, our method includes a more precise organism depth distribution and mobility estimations based on multiple modalities within a trait that can be fuzzy coded. Thirdly, the method is universal and can be applied outside European waters if traits are coded in the same way.

Bioirrigation. We use here ability to ventilate (24), body mass (14), burrow type (21) and burrow depth (22); optionally, body length and burrow width could also be considered as all these traits are directly involved in bioirrigation[9].

```
irr <- syn.trait(tab = tab, lab = lab,
                 which.traits = c(24, 14, 21, 22),
                 list.sco = list(c(1:3),
                                 c(1:6),
                                 c(1:3),
                                 c(1:5)),
                 fuz = c(T, T, T, T),
                 fun = "mean",
                 formula = "x1 * x2 * x3 * x4",
                 sco.scale = T,
                 trait.scale = T,
                 index.scale = T)

sort(irr, decreasing = T)[1:30]

          Upogebia pusilla           Alitta succinea        Glycera tridactyla
                1.00000000                0.90000000                0.90000000
      Hediste diversicolor             Nereis zonata              Mya arenaria
                0.80000000                0.75000000                0.58333333
```

```
  Leptosynapta inhaerens           Nephtys hombergii    Malacoceros fuliginosus
              0.45000000                  0.45000000                 0.33333333
    Notomastus profundus       Perinereis cultrifera         Thracia phaseolina
              0.30000000                  0.30000000                 0.30000000
      Amphiura stepanovi         Corophium volutator          Lagis neapolitana
              0.25000000                  0.25000000                 0.25000000
      Magelona mirabilis       Terebellides stroemii                 Scolelepis
              0.20000000                  0.20000000                 0.19444444
   Prionospio maciolekae     Heteromastus filiformis            Polydora cornuta
              0.15000000                  0.13333333                 0.12500000
        Pygospio elegans         Arcuatula senhousia            Melinna palmata
              0.12500000                  0.11111111                 0.11111111
              Ampelisca     Dipolydora quadrilobata         Cerastoderma glaucum
              0.11111111                  0.10000000                 0.08333333
              Capitella               Spio decorata Streblospio gynobranchiata
              0.08333333                  0.08333333                 0.08333333
...
```

References

1. Chessel, D., Dufour, A. B. & Thioulouse, J. The ade4 package-I-One-table methods. *R News* **4**(1), 5–10, https://journal.r-project.org/articles/RN-2004-002/ (2004).
2. Dray, S., Dufour, A. B. & Chessel, D. The ade4 package-II: Two-table and K-table methods. *R News* **7**(2), 47–52, https://journal.r-project.org/articles/RN-2007-019 (2007).
3. Chevene, F., Doleadec, S. & Chessel, D. A fuzzy coding approach for the analysis of long-term ecological data. *Freshw. Biol*. **31**(3), 295–309, https://doi.org/10.1111/j.1365-2427.1994.tb01742.x (1994).
4. Beauchard, O., Mestdagh, S., Koop, L., Ysebaert, T. & Herman, P. Benthic synecology in a soft sediment shelf: habitat contrasts and assembly rules of life strategies. *Mar. Ecol. Prog. Ser*. **682**, 31–50, https://doi.org/10.3354/meps13928 (2022).
5. Kristensen, E. *et al*. What is bioturbation? The need for a precise definition for fauna in aquatic sciences. *Mar. Ecol. Prog. Ser*. **446**, 285–302, https://doi.org/10.3354/meps09506 (2012).
6. Beauchard, O. *et al*. Assessing sea floor functional biodiversity and vulnerability. *Mar. Ecol. Prog. Ser*. **708**, 21–43, https://doi.org/10.3354/meps14270 (2023).
7. François, F., Poggiale, J. C., Durbec, J. P. & Stora, G. A new approach for the modelling of sediment reworking induced by a macrobenthic community. *Acta Biotheor* **45**, 295–319, https://doi.org/10.1023/A:1000636109604 (1997).
8. Queirós, A. M. *et al*. A bioturbation classification of European marine infaunal invertebrates. *Ecol. Evol*. **3**, 3958–3985, https://doi.org/10.1002/ece3.769 (2013).
9. Kristensen, E. & Kostka, J. E. Macrofaunal burrows and irrigation in marine sediment: Microbiological and biogeochemical interactions. in *Coastal and Estuarine Studies* (eds. Kristensen, E., Haese, R. R. & Kostka, J. E.) vol. 60 125–157 (American Geophysical Union, Washington, D. C., 2005). https://doi.org/10.1029/CE060
